# Understanding the Effect of Ozone on *Listeria monocytogenes* and Resident Microbiota of Gorgonzola Cheese Surface: A Culturomic Approach

**DOI:** 10.3390/foods11172640

**Published:** 2022-08-31

**Authors:** Felice Panebianco, Selene Rubiola, Chiara Buttieri, Pierluigi Aldo Di Ciccio, Francesco Chiesa, Tiziana Civera

**Affiliations:** Department of Veterinary Sciences, University of Turin, Largo Braccini 2, Grugliasco, 10095 Turin, Italy

**Keywords:** cheese, culturomics, food safety, *Listeria monocytogenes*, MALDI-TOF MS, ozone

## Abstract

The occurrence of *Listeria monocytogenes* on Gorgonzola cheese surface was reported by many authors, with risks arising from the translocation of the pathogen inside the product during cutting procedures. Among the novel antimicrobial strategies, ozone may represent a useful tool against *L. monocytogenes* contamination on Gorgonzola cheese rind. In this study, the effect of gaseous ozone (2 and 4 ppm for 10 min) on *L. monocytogenes* and resident microbiota of Gorgonzola cheese rind stored at 4 °C for 63 days was evaluated. A culturomic approach, based on the use of six media and identification of colonies by MALDI-TOF MS, was used to analyse variations of resident populations. The decrease of *L. monocytogenes* was less pronounced in ozonised rinds with final loads of ~1 log CFU/g higher than controls. This behaviour coincided with a lower maximum population density of lactobacilli in treated samples at day 28. No significant differences were detected for the other microbial determinations and resident microbiota composition among treated and control samples. The dominant genera were *Candida*, *Carnobacterium*, *Staphylococcus*, *Penicillium*, *Saccharomyces*, *Aerococcus*, *Yarrowia*, and *Enterococcus*. Based on our results, ozone was ineffective against *L. monocytogenes* contamination on Gorgonzola rinds. The higher final *L. monocytogenes* loads in treated samples could be associated with a suppressive effect of ozone on lactobacilli, since these are antagonists of *L. monocytogenes*. Our outcomes suggest the potential use of culturomics to study the ecosystems of complex matrices, such as the surface of mould and blue-veined cheeses.

## 1. Introduction

*Listeria monocytogenes* is a well-known Gram-positive foodborne pathogen responsible for human listeriosis. Based on the last European Union summary report, listeriosis was the fifth most reported zoonosis in 2020, with 1876 confirmed invasive human cases that led to 780 hospitalisations and 167 deaths [1]. The risk of listeriosis is particularly linked to the consumption of ready-to-eat (RTE) products, for which food safety criteria are prescribed in the Regulation (EC) n. 2073/05 and amendments [2,3]. Several listeriosis outbreaks caused by the consumption of dairy products have occurred worldwide [4,5]. It is well-known that this pathogen can survive and grow in several cheese types during storage, with a particular affinity for blue-veined and mould cheeses, such as Camembert, Danish blue, Brie, Gorgonzola, etc. [6,7].

Gorgonzola is a traditional, blue-veined protected designation of origin (PDO) cheese produced in Northern Italy using cow’s milk inoculated with various thermophilic cultures, yeasts, and *Penicillium* spp. [7]. Several studies reported the occurrence of *L. monocytogenes* on the surface of Gorgonzola cheese, mostly linked to environmental contamination [8,9,10]. For this reason, the rind was declared inedible by the Gorgonzola Consortium [7]. Cutting procedures may be responsible for the dispersion of this foodborne pathogen into the inner parts of Gorgonzola cheese, posing a serious risk to consumers [11]. In this scenario, finding effective decontamination strategies is a major challenge for cheese makers. Ideally, a valid decontamination process should leave no residue in the food, should be inexpensive, and should not reduce the nutritional or chemical characteristics of the product. The main strategies proposed to counteract the contamination of *L. monocytogenes* in Gorgonzola cheese production are essentially based on biological control by using antagonistic lactic acid bacteria [7,12]. Other antimicrobial approaches include physical treatments of the rind, such as infrared, high-pressure processing (HPP), and high-pressure water spray washing [13,14,15]. 

Among the novel technologies to control undesirable microorganisms in the food sector, the application of aqueous or gaseous ozone may be one of the most promising tools. Ozone, in fact, is a broad-spectrum antimicrobial with low environmental impact and does not leave residues on surfaces or in finished products [16,17,18,19]. Several studies showed the effect of ozone against *L. monocytogenes* in planktonic form or in biofilm state as well as its potential in controlling the pathogen in food [20,21,22,23,24,25]. The use of ozone in direct contact with different foods has been allowed in the USA since 2001. In the European Union, there is still a lack of specific and uniform legislation on its application [19,26]. In Italy, the Ministry of Health approved the use of ozone for disinfection of cheese ripening rooms but not in direct contact with products [22,27]. However, an application of low-dose ozone in direct contact with whole cheeses at the end of the ripening process may be useful to improve the control of surface contamination by *L. monocytogenes* and to prevent the transfer of this pathogen inside the products during cutting procedures. In this regard, it would be interesting to study the behaviour of *L. monocytogenes* on rinds during extended storage at refrigeration temperatures after a preliminary ozone exposure as well as the impact of ozone treatment on resident cheese microbiota.

To study the evolution of microbiota, culturomics is one of the most interesting approaches. This method is based on the use of multiple culture conditions and the application of matrix-assisted laser desorption–ionization time of flight mass spectrometry (MALDI-TOF MS) for the rapid identification of bacterial genera and species. This approach has led to a complete characterization of bacterial populations of the human gut microbiota as well as to the discovery of new microorganisms associated with humans [28]. Culturomics appears promising also for the characterization of food microbiota, given the reliability of MALDI-TOF MS in identification of food-related bacteria [29,30]. Recently, culturomics and MALDI-TOF MS identification were successfully applied to characterise the microbiota of raw milk [31], the spoilage bacteria of poultry meat [32], the microbial communities of wild boar meat [33], the bacteria of chilled vacuum-packaged lamb meat [34], and the microbiota of the artisanal soured cream Staka [35]. Previous studies showed that the surface of Gorgonzola cheese is a complex and heterogeneous matrix due to the presence of several microbial populations that coexist during storage [36,37,38]. In view of this, a culturomic approach based on routine culturing of microorganisms in different media and subsequent identification of colonies by MALDI-TOF MS appears as a promising way to completely discover the populations typifying this complex environment.

Since literature data on the effect of ozone against *L. monocytogenes* and its impact on resident microbiota of Gorgonzola cheese surface are still scarce, the aims of the current study were: (i) to evaluate the effect of this nonthermal decontamination process (gaseous ozone) on survival and growth of *L. monocytogenes* in the presence of resident microbiota on surface of ripened Gorgonzola cheese during storage at 4 °C for 63 days and (ii) to estimate the impact of ozone treatment on resident cheese microbiota by using a culturomic approach.

## 2. Materials and Methods

### 2.1. Gorgonzola Rind Samples

Gorgonzola rinds from ripened cheeses (60 days) of the same production batch provided by a local dairy industry (Piedmont, Italy) were used in this study. Rinds were transported in cool containers to the laboratory of the Food Safety Unit, Department of Veterinary Sciences, University of Turin, Italy. Firstly, rind samples were checked for the presence of *L. monocytogenes* according to the ISO 11290-1 2017. Briefly, 25 g of each rind randomly selected were suspended (10% *w*/*v*) in half-Fraser broth (Sifin, Berlin, Germany) and incubated at 30 °C for 24 h. Afterwards, 0.1 mL of this first enrichment were inoculated on plates of Oxoid Chromogenic Listeria Agar (OCLA, Chromogenic Listeria Agar Base + OCLA selective supplement + Brilliance^TM^ Listeria differential supplement; Oxoid, Basingstoke, UK) and Palcam Agar (Palcam Agar Base + Palcam selective supplement; Oxoid), incubated at 37 °C for 48 h. A subculture of 0.1 mL from the half-Fraser (Sifin) broth was performed in 10 mL of Fraser broth (Sifin). After 24 h at 37 °C, 0.1 mL of the culture from the second enrichment were plated on OCLA (Oxoid) and Palcam (Oxoid) agar. Plates were incubated at 37 °C for 48 h and then checked for typical *L. monocytogenes* colonies. Absence of typical colonies on selective media confirmed that rinds were ready for the challenge test trials.

### 2.2. Inoculum Preparation

Three *L. monocytogenes* strains, completely sequenced (whole genome sequencing) and previously isolated from Gorgonzola cheese, were used in this study (Table 1). 

All strains were obtained from the bacterial culture collection of the Department of Veterinary Sciences, University of Turin, Italy. Before the experiment, strains were reactivated from the frozen storage (−20 °C; 80% Brain Heart Infusion broth (BHI, Oxoid); 20% glycerol) in BHI (Oxoid) broth at 37 °C for 48 h. Strains were then cultured in BHI (Oxoid) broth at 4 °C for 10 days in order to habituate cells to the conditions of the subsequent challenge test [12] and diluted to obtain the desired concentration (5–6 log CFU/mL).

### 2.3. Inoculation of Rind Samples and Ozone Treatments

Rinds were portioned in sterile conditions to obtain samples of 5 g (~5.0 × 5.0 cm). All samples were inoculated on the surface with a cocktail of the three *L. monocytogenes* strains to reach a final target level ranging between 3–4 log CFU/g. Inoculation was carried out by adding an appropriate amount (0.1 mL) of diluted bacterial culture obtained as described above (see Section 2.2) and distributing it uniformly over the entire surface of the rind samples. Inoculated rind portions were then divided in three equally numerous groups: O2, O4, and control. Samples of the group O2 were treated with gaseous ozone at 2 ppm (10 min), whereas samples from the O4 group were treated with 4 ppm (10 min) of gaseous ozone. The concentrations and the treatment times used in our experiment were set based on already published data that showed a promising anti-*Listeria* effect of gaseous ozone at concentrations between 1 and 4 ppm as well as a negligible action in terms of fat oxidation in Gorgonzola cheese [21,39,40,41]. Control samples were not subjected to ozone exposure. Ozone treatments were performed at room temperature in an ozone-inert plexiglass chamber connected to an ozone generator (Model-LF5; Biofresh Group Ltd., Northumberland, UK). The injection of ozone in the chamber was controlled by an ozone analyser (UV-100, EcoSensor, Santa Fe, NM, USA). A fan was placed inside to ensure a uniform distribution of the gas in the chamber. The relative humidity (RH) was maintained at levels ≥ 90% by placing water-filled containers at the bottom of the chamber. It has been proven, in fact, that the bactericidal effect of gaseous ozone increases with high RH (optimum at 90–95%) [42]. Treated (O2, O4) and control samples were stored at 4 °C to simulate the standard retail conditions of cheese. Temperature and RH (%) levels during the ozone treatments and the subsequent storage of samples were monitored with data loggers (Testo 174 H, Testo AG, Lenzkirchen, Germany).

### 2.4. Microbiological Analysis and Determination of pH/a_w_

In order to quantify the microbial population persisting on inoculated rinds, a random selection of three samples for each series (control, O2, O4) was removed from the incubator at the beginning (day 0; immediately after the ozone treatments for samples O2 and O4) and after 3, 7, 14, 21, 28, 35, 42, 49, 56, and 63 days of storage at 4 °C for a total of 99 samples during the entire experiment (33 for each series). Similarly, pH and *a_w_* measurements were performed in triplicate at each sampling point.

For microbiological analysis, rind samples were suspended (10% *w*/*v*) in sterile physiological saline peptone (PS; 0.85% NaCl + 0.1% Bacto-Peptone) and homogenised for 30 s (Stomacher 400 Circulator; Seward, London, UK), and then, 10-fold dilutions were prepared in tubes containing 9 mL of PS. In detail, the following enumerations were performed: (a) *L. monocytogenes* on OCLA (Oxoid), incubated at 37 °C for 48 h; (b) total viable count on Plate Count Agar (Oxoid), incubated at 30 °C for 48–72 h; (c) enterococci on Slanetz Bartley Agar (Biolife, Milan, Italy), incubated at 37 °C per 48 h; (d) coagulase-positive staphylococci on Baird Parker Agar (Oxoid) supplemented with Egg Yolk Tellurite emulsion (Oxoid), incubated at 37 °C for 48 h; (e) mesophilic lactobacilli on De Man Rogosa and Sharp (MRS) Agar (Oxoid), incubated anaerobically at 30 °C for 48 h; (f) mesophilic cocci on M17 Agar (Oxoid), incubated anaerobically at 30 °C for 48 h; (g) yeasts and moulds on Potato Dextrose Chloramphenicol Agar (Conda, Madrid, Spain), incubated at 30 °C for 48–72 h. 

In addition, pH was measured (Crison 507; Crison Instruments, Barcelona, Spain) after stirring the rind samples (5 g) with deionised water (25 mL), while *a_w_* was determined with a water activity meter (Aqualab Series 3 TE; Decagon Devices, Pullman, Washington, DC, USA).

### 2.5. MALDI-TOF MS Identification and Culturomic Analysis

Changes in resident microbiota composition during storage were assessed with a culturomic approach based on the identification of colonies by MALDI-TOF MS. At each sampling point, three colonies for each morphological type, selected considering size, profile, elevation, boundary, and colour [43], were picked from plates with the highest dilution factor and a number of colonies ≤ 300 of different media (except OCLA) used for microbiological analyses, for a total of 1061 colonies during the entire experiment. Colonies were spotted on 96-well MALDI Target Plate (MSP 96 target; Bruker Daltonics, Bremen, Germany) and overlaid with 1 μL of 70% formic acid solution. Air dried spots were then covered with 1 μL of a matrix solution (α-cyano-4-hydroxycinnamic acid; Bruker Daltonics). Mass spectra were acquired in linear positive mode (mass range 2000–20,000 Da) with Microflex LT MALDI-TOF mass spectrometer (Bruker Daltonics) controlled by FlexControl software 4.1.9 (Bruker Daltonics) and associated with MALDI BioTyper database (Bruker Daltonics). According to the manufacturer indications, score values between 2.00–3.00 indicated a high-confidence identification (identification at species level), score values between 1.70–1.99 were considered as low-confidence identifications (identification at genus level), while score values between 0.00–1.69 indicated no identification possible. To minimise the risk of underestimating subdominant strains, in addition to the total count for each medium, a differential count for each genus identified was performed including all plates with a number of colonies ≤ 300.

### 2.6. Statistical Analyses and Graphing

Data of microbial enumerations, pH, and *a_w_* were analysed with a two-way ANOVA followed by a Tukey’s multiple comparison test (*p* < 0.05; GraphPad Prism version 9.0.0, GraphPad Software, San Diego, CA, USA) to detect significant differences between control and treated (O2 and O4) samples. The relative abundances (%) of microbial genera identified (MALDI-TOF scores between 1.70–1.99) were calculated (Microsoft Excel 2019; Microsoft Corporation, Redmond, WA, USA) by the counted colonies of each genus by the total detectable microbial colonies count [44]. Within each genus, the percentage of microorganisms identified at species level (MALDI-TOF scores ≥ 2.00) over the whole experiment was calculated (Microsoft Excel 2019). Graphs were created with GraphPad Prism version 9.0.0 and Microsoft Excel 2019.

## 3. Results

### 3.1. Behaviour of L. monocytogenes and Resident Populations during Storage

**Figure 1:**
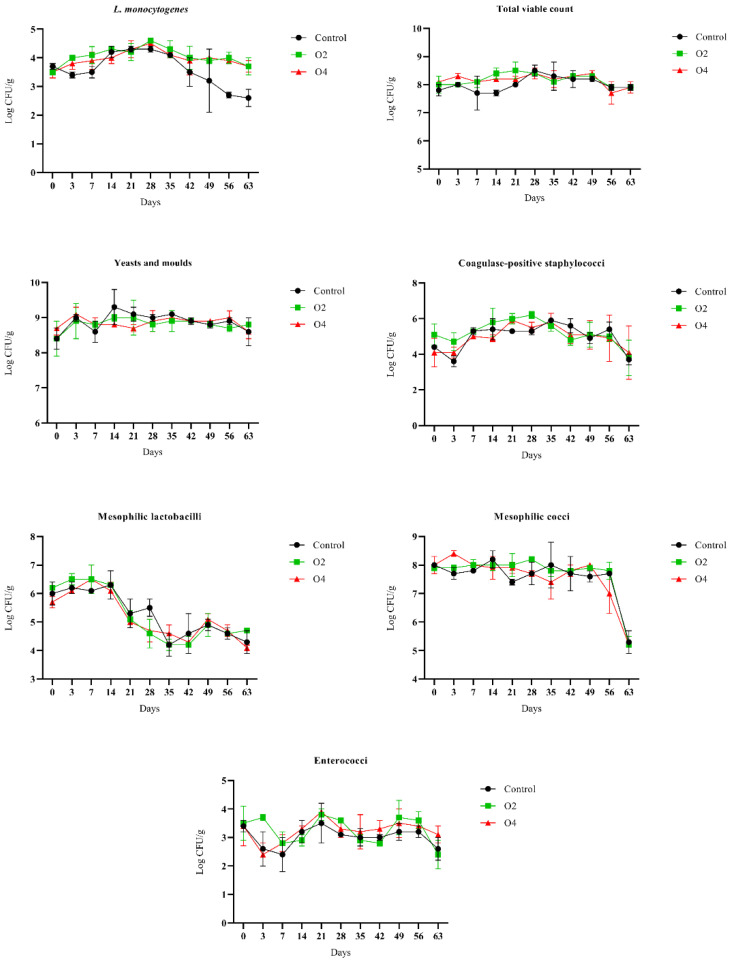
Behaviour of different microbial populations enumerated in control and ozone-treated (O2 and O4) rind samples during storage at 4 °C. Error bars indicate the standard deviation between three replicates.

Outcomes of microbiological enumerations are shown in Figure 1, while detailed data are reported in Table S1.

*L. monocytogenes* grew by about 1 log CFU/g in 28 days in treated (O2 and O4) and control samples. A growth dampening and a subsequent slight decrease was observed, especially in control samples, from day 35. At the end of storage (63 days), the loads were 2.6 ± 0.3, 3.7 ± 0.3, and 3.7 ± 0.2 log CFU/g in control, O2, and O4 samples, respectively. Total viable counts and yeasts and moulds remained stable during the entire storage without significant differences among control and treated samples. Coagulase-positive staphylococci reached the highest loads in 35 days in control samples (5.9 ± 0.1 log CFU/g), in 28 days in O2 samples (6.2 ± 0.2 log CFU/g), and in 21 days in O4 samples (5.9 ± 0.2 log CFU/g). Similar behaviour of enterococci was observed in control and treated samples, with the highest values detected at 21 days of storage (3.5 ± 0.7, 3.8 ± 0.2, and 3.9 ± 0.3 log CFU/g in control, O2, and O4 samples, respectively). For mesophilic lactobacilli, a decrease was detected form day 21 of storage in treated and control samples. At day 28, however, this reduction was more significant in treated samples (loads = 4.6 ± 0.5 and 4.7 ± 0.4 log CFU/g in O2 and O4 samples, respectively) compared to controls (loads = 5.5 ± 0.3 log CFU/g). Mesophilic cocci instead remained stable during the storage in all samples, with a significant decrease detected at the last sampling point (day 63; 5.3 ± 0.4, 5.2 ± 0.3, and 5.2 ± 0.3 for control, O2, and O4 samples).

### 3.2. Changes in pH and a_w_ during Storage

**Figure 2:**
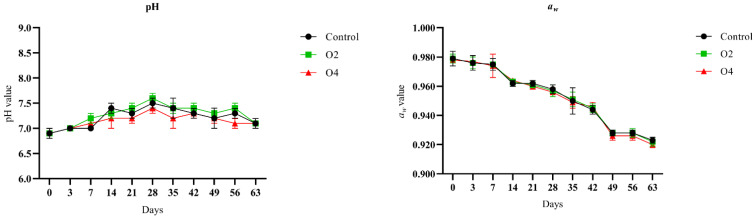
Values of pH and *a_w_* in control and ozone-treated (O2 and O4) rind samples during storage at 4 °C. Error bars indicate the standard deviation between three replicates.

The pH and *a_w_* measurements during storage followed the same pattern for control and treated samples (Figure 2).

A slight increase in pH was observed up to day 28 of storage for all samples (values of 7.5 ± 0.1, 7.6 ± 0.1, and 7.4 ± 0.1 for control, O2, and O4, respectively), followed by a modest decrease until values of ~7.1 at the end of storage. A progressive reduction of *a_w_* was observed, until final values of 0.923 ± 0.002, 0.922 ± 0.003, and 0.920 ± 0.002 for control, O2, and O4 samples, respectively.

### 3.3. Culturomic Analysis and MALDI-TOF MS Identification

#### 3.3.1. Relative Abundances of Microbial Genera

**Figure 3:**
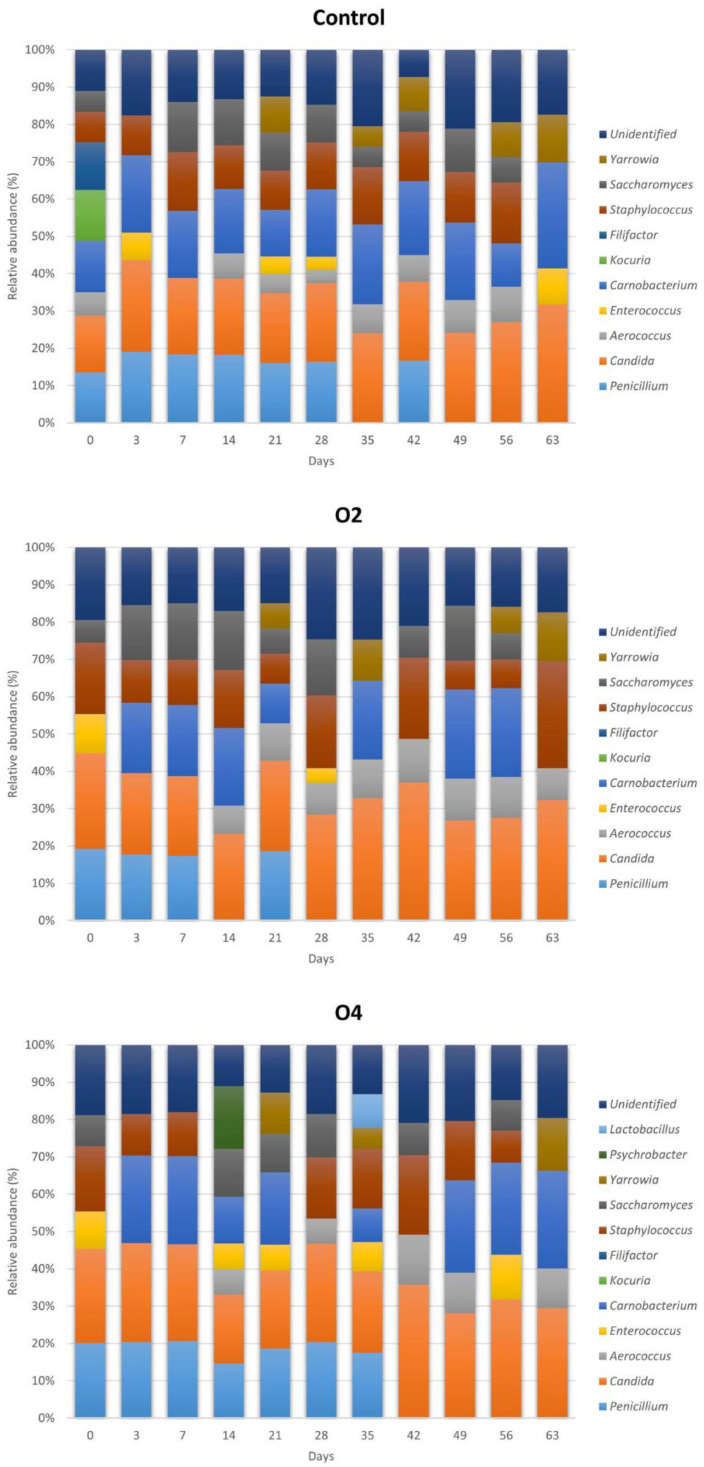
Relative abundances (%) of different microbial genera in control and ozone-treated (O2 and O4) rind samples during storage at 4 °C.

Relative abundances (RA) of microbial genera during the experiment are reported in Figure 3. 

During the entire experiment, the dominant populations in control samples were *Candida* (mean RA = 22.6%), *Carnobacterium* (mean RA = 18.4%), *Staphylococcus* (mean RA = 11.6%), *Penicillium* (mean RA = 10.8%), *Saccharomyces* (mean RA = 7.4%), *Aerococcus* (mean RA = 5.0%), *Yarrowia* (mean RA = 4.2%), *Enterococcus* (mean RA = 2.3%), *Kocuria* and *Filifactor* (mean RA = 1.2%). The dominant genera at the beginning of the experiment (day 0) were *Candida* (15.2%), *Carnobacterium* (13.7%), *Kocuria* (13.7%), *Penicillium* (13.5%), *Filifactor* (12.9%), *Staphylococcus* (8.1%), *Aerococcus* (6.2%), and *Saccharomyces* (5.5%). *Kocuria* and *Filifactor* were detected no further during storage. *Candida*, *Carnobacterium*, *Staphylococcus*, and *Saccharomyces* were detected until the last days of storage, while *Penicillium* was detected until day 42.

In O2 samples, the dominant genera during the entire experiment were *Candida* (mean RA = 27.4%), *Staphylococcus* (mean RA = 13.8%), *Carnobacterium* (mean RA = 12.6%), *Saccharomyces* (mean RA = 9.4%), *Aerococcus* (mean RA = 7.2%), *Penicillium* (mean RA = 6.6%), *Yarrowia* (mean RA = 3.5%), and *Enterococcus* (mean RA = 1.3%). The dominant genera at the beginning of the experiment (day 0) were *Candida* (25.6%), *Penicillium* (19.2%), *Staphylococcus* (19.2%), *Enterococcus* (10.5%), and *Saccharomyces* (6.0%). *Carnobacterium*, *Aerococcus*, and *Yarrowia* were detected during the next days of storage and remained relatively stable until the last days. *Enterococcus* was detected only one time during the rest of the experiment (day 28). *Penicillium* was detected until day 21, while *Candida*, *Staphylococcus*, and *Saccharomyces* remained stable until the end of storage.

In O4 samples, the dominant genera during the entire experiment were *Candida* (mean RA = 26.4%), *Carnobacterium* (mean RA = 14.9%), *Penicillium* (mean RA = 12.0%), *Staphylococcus* (mean RA = 10.8%), *Saccharomyces* (mean RA = 5.5%), *Aerococcus* (mean RA = 4.4%), *Enterococcus* (mean RA = 4.0%), *Yarrowia* (mean RA = 2.8%), *Psychrobacter* (mean RA = 1.5%), and *Lactobacillus* (mean RA = 0.8%). The dominant genera at the beginning of the experiment (day 0) were *Candida* (25.3%), *Penicillium* (20.1%), *Staphylococcus* (17.4%), *Enterococcus* (10.0%), and *Saccharomyces* (8.4%). *Carnobacterium*, *Aerococcus*, and *Yarrowia* were detected during the next days of storage and remained relatively stable until the end of storage. *Penicillium* was detected until day 35, while *Candida*, *Staphylococcus*, and *Saccharomyces* remained stable until the end of storage.

#### 3.3.2. Percentage of Colonies Identified at Species Level

**Figure 4:**
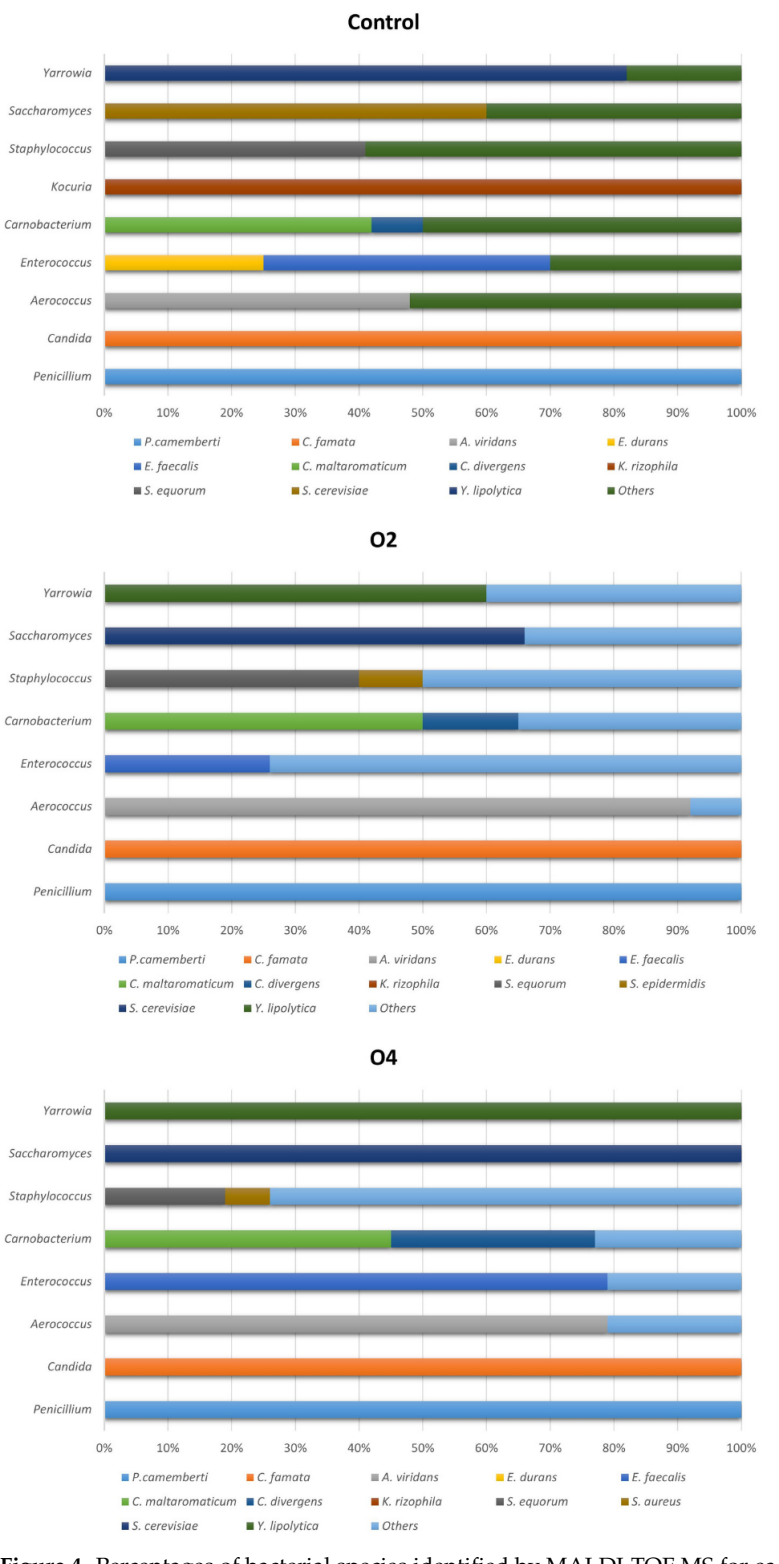
Percentages of bacterial species identified by MALDI-TOF MS for each genus in control and ozone-treated (O2 and O4) rind samples during the experiment.

Percentages of microbial species identified by MALDI-TOF MS for each genus during the entire experiment are reported in Figure 4. 

All colonies belonging to *Penicillium* and *Candida* genera were identified as *P. camemberti* and *C. famata* (also known as *Debaryomyces hansenii*) in treated and control samples. Bacteria of the genus *Aerococcus* were often identified as *A. viridans* (48, 92, and 79% in control, O2, and O4 samples, respectively). *Enterococcus* were identified as *E. faecalis* (45, 26, and 79% in control, O2, and O4 samples, respectively), while *E. durans* were detected only in control samples (25%). Colonies of the genus *Carnobacterium* were identified as *C. maltaromaticum* (47, 50, and 45% in control, O2, and O4 samples, respectively) and *C. divergens* (9, 15, and 32% in control, O2, and O4 samples). Regarding the genus *Kocuria*, detected only in control samples, all colonies were identified as *K. rizophila*. In the genus *Staphylococcus*, *S. equorum* was identified in all samples (41, 40, and 19% in control, O2, and O4 samples, respectively). Colonies of *S. epidermidis* were identified in O2 samples (10%), while *S. aureus* was detected in O4 samples (7%). Colonies belonging to genus *Saccharomyces* were identified as *S. cerevisiae* in most cases (60, 66, and 100% in control, O2, and O4 samples, respectively), while *Yarrowia* genus was dominated by *Y. lipolytica* (82, 60, and 100% in control, O2, and O4 samples, respectively).

## 4. Discussion

Given the economic importance of Gorgonzola cheese and its worldwide commercialization, it is crucial to control *L. monocytogenes* contamination in this Italian dairy product. If present, *L. monocytogenes* contamination in Gorgonzola cheese is often limited to the product surface [7,8,13,36]. However, it is not possible to exclude the transferring of the pathogen to the inner portions of cheese during cutting procedures at production and retail premises [11]. In light of this, there is an urgent need to invest in novel strategies to prevent and contrast *L. monocytogenes* contamination on Gorgonzola cheese rind. In the present study, we applied ozone gas on Gorgonzola cheese (rind samples artificially inoculated and stored at 4 °C) at the end of the ripening process (60 days) for evaluating its effect against *L. monocytogenes*. In addition, the impact of ozone treatment on resident microbiota was investigated. In nature, *L. monocytogenes* contamination of food may involve more than one strain, so a simple inoculation model composed of three dairy-related strains was used in this experiment. In setting up effective food treatments with ozone, it is crucial to determine the concentration to be used and how long products should be exposed to the gas. In order to obtain an inhibiting effect on *L. monocytogenes* without substantial changes of product characteristics and avoiding the application of high ozone levels dangerous for the operators [19], we analysed data from the literature. Studies demonstrated that ozone in a gaseous state could inactivate *L. monocytogenes* in vitro by applying low concentrations combined with short treatment times. In the study of Morandi et al. [39], the application of gaseous ozone at 4 ppm for 8 min led to a reduction in the number of *L. monocytogenes* (1–1000 CFU/g) of more than 99% on ALOA agar. Furthermore, lower ozone concentrations can be effective; Robbins et al. [40] reported a complete inactivation of unattached *L. monocytogenes* Scott A (reduction = 8.29 log CFU/mL) inoculated in PPB after exposure to 0.25 ppm of ozone for 3 min. With a lowest ozone concentration (0.05 ppm), Bigi et al. [22] observed a high resistance of *L. monocytogenes* in vitro until 6 h treatment, with a significant reduction detected only after 24 h treatment. In food, the effectiveness of ozone gas seems linked to the type of products, but generally, even in this case, low concentrations and short exposure times appear effective. In beef, treatments with ozone pulses (280 mg O3 m^−3^) for 5 and 10 min every 30 min for 5 h reduced the loads of inoculated *L. monocytogenes* (10^2^ CFU/g tissue^−1^) to values below the detection limit [25]. A study conducted by Muthukumar and Muthuchamy [45] demonstrated that ozone at 33 mg/min for 9 min in the gaseous phase inactivated 2 × 10^6^ CFU/g of *L. monocytogenes* on chicken samples. Wani et al. [41] observed a 1-log reduction of *L. monocytogenes* on spinach leaves after 10 min at 1 ppm of gaseous ozone. In regard to cheese, Morandi et al. [39] conducted a study to evaluate the effect of ozonisation (4 ppm for 8 min) against *L. monocytogenes* on different cheese products, such as Ricotta Salata, Taleggio, and Gorgonzola, at three different ripening times and two inoculum levels (1–100 CFU/g and 100–1000 CFU/g). Ozone exposure reduced the loads below 10 CFU/g, and no growth was observed after 7 days of storage in Ricotta Salata cheese. Treatments were effective also in short-ripened Taleggio and Gorgonzola cheese; the logarithmic reduction was less pronounced but still observed in the more ripened cheeses. Analysis of product characteristics revealed a negligible effect of treatments on cheeses, since a lower presence of lipolytic compounds and oxidation products was detected in the layer just below the rind. Considering the above literature data, we decided to apply ozone gas at 2 and 4 ppm for 10 min, hypothesising a possible application on whole Gorgonzola forms at the end of ripening to counteract *L. monocytogenes* surface contamination, thus reducing the risk of pathogen transfer into the cheese after cutting operations. Furthermore, we studied the behaviour of *L. monocytogenes* on ozone-treated rinds stored at 4 °C to simulate the commercialisation and storage of the product at a large-scale retail level. Contextually, we enumerated and studied the microbial populations typical of this matrix in order to evaluate the possible effect of ozone treatment on the resident microbiota of Gorgonzola cheese. 

In our study, *L. monocytogenes* showed comparable growth dynamics in control and ozonised samples until day 28 of storage at 4 °C, with an increase of about 1 log CFU/g in 28 days. From this point, we observed a decrease of *L. monocytogenes* in both control and ozonised samples (see Section 3.1, Figure 1, and Table S1). This decline was more evident in treated samples, and a significant difference in terms of *L. monocytogenes* loads was detected at the end of storage (day 63). In control samples, in fact, *L. monocytogenes* was detectable with loads of 2.6 ± 0.3 log CFU/g, while the counts in ozonised samples (3.7 ± 0.3 and 3.7 ± 0.2 log CFU/g in O2 and O4 samples, respectively) were significantly (*p* < 0.05) higher. An explanation of this phenomenon could be related to the effect of ozone treatment on other microbial populations that usually act as antagonists against *L. monocytogenes*. In fact, in samples treated with gaseous ozone loads of mesophilic lactobacilli were significantly lower than control samples at day 28, exactly when *L. monocytogenes* was starting its decline phase (see Section 3.1, Figure 1, and Table S1). It is well-known that lactic acid bacteria (LAB) can contrast growth of *L. monocytogenes* in cheese through several mechanisms, such as competition for nutrients, reduction in pH values, and production of antimicrobial compounds (organic acids, hydrogen peroxide, diacetyl, bacteriocins) [46,47,48,49]. Although loads of lactobacilli in control and ozone-treated samples were significantly different only at day 28, the differences in maximum population density (N_max_) at this sampling point may have affected the behaviour of *L. monocytogenes*. Indeed, according to the well-known Jameson effect, when dominant populations (often represented by LAB in food) reach the maximum population density, *L. monocytogenes* stops its growth [50,51,52,53]. We hypothesise that the lower N_max_ reached by mesophilic lactobacilli in ozone-treated samples at day 28 influenced the subsequent behaviour of *L. monocytogenes*, resulting in a less pronounced decline of the pathogen and slightly higher loads at the end of storage in O2 and O4 samples. The potential effect of ozone on LAB behaviour is supported by other scientific evidence. Ozone, in fact, is considered one of the most powerful disinfectants against LAB in the food industry [54]. In a study conducted on vacuum-packaged shucked mussels [55], ozonation in aqueous solution (1 mg/L for 60 and 90 min) caused a significant effect on LAB growth (reduction by 0.3–0.8 log cycles) during refrigerated storage even if no action was observed immediately after treatment. In addition, the population densities of LAB at the end of storage were always lower in treated compared to control samples. In a work conducted by Segat et al. [56], mozzarella cheese cooled with ozone pre-treated (2 mg/L) water showed significantly lower LAB loads than products cooled with non-ozonated water during 21 days of refrigerated storage. A significant effect of gaseous ozone (276–286 mg O3 m^−3^) in reducing the loads of LAB was reported in a recent study conducted on beef [25]. Even though we must underline that the potential effect of ozone on lactobacilli in our experiment is just a hypothesis, the role of ozone treatments on LAB flora should be further investigated. While it could be useful when LAB act as spoilage agents, it could also be harmful. LAB are the dominant population in many fermented foods and their suppression could have deleterious effects on the sensory characteristics of products, as well as favouring the proliferation of pathogenic microorganisms normally counteracted by LAB. Similar to our findings, the limited efficiency of ozone in decontamination of food surfaces was also reported in other studies. Werlang et al. [57] evaluated the effect of gaseous ozone treatment (5.0 ppm during two periods of 4 h interspersed by 6 h of chilling) on microbiological quality of pig carcasses. After ozone application, no significant decrease in the number of carcasses positive for *Listeria* was detected. Segat et al. [56] evaluated the effect of ozone on mozzarella cheese packaged with ozonated water (2 mg/L), treated with ozonated water (2, 5, and 10 mg/L for 60 min), and exposed to gaseous ozone (10, 20, and 30 mg/m^3^) at different treatment times. All treatments were ineffective in significantly reducing the loads of spoilage bacteria of cheese surface. The authors explained how organic compounds may have limited the effectiveness of ozone in some cases, with a subsequential low impact of treatments on microorganisms present on cheese surface. The influence of organic materials on the antimicrobial action of ozone was also demonstrated by de Candia et al. [23], who observed how organic matter (meat extract) reduced the efficacy of ozone treatments against *L. monocytogenes* on polystyrene. These data together with our results suggest that the effectiveness of ozone is closely dependent on the nature and complexity of products to be treated, as well as on the presence of organic components that could limit its antimicrobial action. Therefore, the treatments to be implemented require a thorough preliminary study of the food. Probably, in the case of complex matrices, it may be necessary to apply higher concentrations of ozone to achieve an appropriate antimicrobial effect. Regarding other microbial populations, the action of gaseous ozone at these low concentrations did not lead to significant changes in microbial counts. Therefore, the application of this technology to sanitise the environments is not expected to cause substantial modifications of microbial loads in the finished products and could also be applied in ripening rooms with cheeses at the end of storage. Certainly, in view of the possible negative effects of ozone on fat and the potential concerns for operators due to its toxicity at high concentrations [19,22], treatments should have a major positive impact to compensate for the possible adverse effects.

The culturomic approach used in the present study allowed to understand the difference in bacterial genera and species abundances among control and ozonised samples during storage at 4 °C (see Section 3.3 and Figure 3 and Figure 4). In the past years, several authors studied the microbiota of Gorgonzola rinds, demonstrating the complexity and heterogeneity of this matrix [36,37,38]. In our study, *Candida* was detected as the dominant yeast genus in both control and treated samples, and all colonies were identified at species level as *C. famata* (also known as *Debaryomyces hansenii*). These findings are in accordance with the study of Cocolin et al. [36] that investigated the microbiota of Gorgonzola rinds and maturing shelf swabs in five different maturing cellars in Italy, identifying only *D. hansenii* as the yeast species. Other detected yeast genera in our study were *Yarrowia* and *Saccharomyces*, dominated by the species *Y. lipolytica* and *S. cerevisiae*. These two species are often part of the yeast population of fermented dairy products and they were isolated from Brazilian Gorgonzola-type cheese [58]. Regarding moulds, the detection of *Penicillium* spp. appears obvious and justifiable since *P. camemberti* and *P. roqueforti* are used as starter cultures for blue cheeses production. Among bacterial genera, we detected *Carnobacterium*, *Staphylococcus*, *Aerococcus*, and *Enterococcus* as dominants in all samples. *Kocuria* and *Filifactor* were detected only in control samples, while *Psychrobacter* and *Lactobacillus* only in O4 samples. Even for bacterial genera, our outcomes are in agreement with previous studies that reported the presence of *Carnobacterium*, *Staphylococcus*, *Enterococcus*, and *Psychrobacter* in Gorgonzola rinds [36,37]. Our study provides new information among the dominant species of *Carnobacterium* (*C. maltaromaticum* and *C. divergens*) and confirms the data published by Correa et al. and Fontana et al. [37,58], which reported *S. equorum* as the dominant species among the genus *Staphylococcus* and the presence of *E. faecalis* among the genus *Enterococcus*. The occurrence of the foodborne pathogen *S. aureus* in some samples of the O4 group is certainly remarkable. A new finding with respect to these previous studies was the detection of *Aerococcus* (mostly identified as *A. viridans*) in many of the samples analysed. Analyses of microbiota variations during storage (see Section 3.3 and Figure 3) emphasise that the evolution of different populations over time was similar among control and ozonised samples. Regarding yeasts, *Candida* and *Saccharomyces* were present constantly from the beginning to the end of storage in all samples, while *Yarrowia* became detectable from day 21 and remained stable until the end of the experiment. *Penicillium* followed the same pattern in all samples, becoming undetectable over time. *Aerococcus*, *Carnobacterium*, and *Staphylococcus* remained present until the end of storage even if with some difference in terms of relative abundance. Besides the positive elements, the limitations of culturomics for the study of microbiota in complex matrices must also be emphasised. In the case of Gorgonzola cheese rind, as an example, a high inter-sample variability due to a non-uniform distribution of microorganisms on the product surface could be detected. In sporadic cases, we may not find some microbial genera in certain samples and detect them in others. This may explain the disappearance and reappearance of *Penicillium* in control and O2 samples at some points (see Figure 3). However, in a sampling plan of 63 days, this event occurred sporadically and constituted a minor variable for our experiment that was minimised by calculating the mean relative abundances (see Section 3.3.1) for each microbial genus during the entire storage of products. Our objective, in fact, was to highlight marked differences, e.g., the complete suppression of one or more microbial genera during the entire experiment, in resident microbiota composition between ozone-treated and control samples. The method used for the selection of colonies, even if performed in an accurate and systematic way, may depend on the ability of operators, is susceptible to some minor errors, and could represent another limitation factor. To reduce this variable, we identified three colonies for each selected type at each sampling point (see Section 2.5) in order to ensure that all colonies visualised as morphologically identical actually belonged to the same genus and/or species. Furthermore, the use of more and product-adapted culture media is recommended for culturomic studies to avoid the underestimation of certain microbial populations. In our study, we used specific media chosen after a thorough literature review and based on the microbial populations expected in this type of matrix [36,37,38,58]. Even considering these limitations, in light of the results of the culturomic analysis supported by the outcomes of microbial enumerations, characterised by few significant variations and low standard deviations among samples, we can state that ozone exposure did not result in substantial modifications of resident microbiota of Gorgonzola cheese surface during refrigerated storage. The results obtained and the comparability of our findings with the other surveys previously reported suggest that the method applied was sufficient to minimise the variables and demonstrate the limited effect of gaseous ozone on the microbial populations typical of this product.

## 5. Conclusions

This research was carried out to evaluate the behaviour of *L. monocytogenes* on the surface of ripened Gorgonzola cheese after ozone treatment during refrigerated storage. Our study demonstrated that ozone gas treatments at 2 and 4 ppm for 10 min were ineffective against *L. monocytogenes* artificially inoculated on Gorgonzola cheese rinds stored at 4 °C for 63 days. The maximum population density reached by mesophilic lactobacilli was lower in ozone-treated samples. No significant difference among ozonised and control samples were detected for the other microbial enumerations (total viable count, enterococci, coagulase-positive staphylococci, mesophilic cocci, yeasts and moulds). The culturomic analysis based on the identification of colonies with MALDI-TOF MS led to comprehensive data about the effect of ozone on resident cheese microbiota, highlighting no significant difference among ozone-treated and control samples. This approach could be a rapid and efficient tool for studying the microbiota of food even in a complex matrix such as the surface of blue-veined cheeses. 

## Figures and Tables

**Table 1:** Characteristics of *L. monocytogenes* strains used for challenge tests.

Internal ID	NCBI ID ^a^	Isolation Source	Year	Lineage	Serogroup	Sequence Type	Clonal Complex
G40	CFSAN044840	Gorgonzola cheese	2005	II	IIa	325	31
G52	CFSAN044807	Gorgonzola cheese	2004	II	Iia	325	31
G70	CFSAN044814	Gorgonzola cheese	2004	II	Iia	325	31

^a^ ID of the strains in the NCBI database (https://www.ncbi.nlm.nih.gov/; accessed on 15 March 2022).

## Data Availability

Data is contained within the article or Supplementary Material. Further inquiries may be directed to the corresponding author.

## Supplementary Materials

The following supporting information can be downloaded at: https://www.mdpi.com/article/10.3390/foods11172640/s1, Table S1: Counts (Log CFU/g) of different microbial populations in control and ozone treated (O2 and 04) rinds during storage at 4 °C.
