# Supplementary material for: Understanding the Effect of Ozone on Listeria monocytogenes and Resident Microbiota of Gorgonzola Cheese Surface: A Culturomic Approach
Source: Foods. 2022 Aug 31;11(17):2640. doi: 10.3390/foods11172640 (PMC9455919; doi:10.3390/foods11172640)
Supplement: Supplementary file 1 [file foods-11-02640-s001.zip › foods-1845410-supplementary.pdf]

Table S1 Counts (Log CFU/g) of different microbial populations in control and ozone treated (O2 and 04) rinds during storage at 4°C.

Time (days)	*L. monocytogenes*			Total Viable Count			Coagulase-positive staphylococci			Enterococci			Mesophilic lactobacilli			Mesophilic cocci			Yeasts and Moulds		
	Control	O2	O4	Control	O2	O4	Control	O2	O4	Control	O2	O4	Control	O2	O4	Control	O2	O4	Control	O2	O4
0	3,7 ± 0,1	3,5 ± 0,1	3,5 ± 0,2	7,8 ± 0,2	8,0 ± 0,3	8,1 ± 0,1	4,4 ± 0,0	5,1 ± 0,6	4,1 ± 0,8	3,4 ± 0,2	3,5 ± 0,6	3,4 ± 0,7	6,0 ± 0,4	6,2 ± 0,2	5,7 ± 0,2	8,0 ± 0,0	7,9 ± 0,1	8,0 ± 0,3	8,4 ± 0,3	8,4 ± 0,5	8,7 ± 0,2
3	3,4 ± 0,1 a	4,0 ± 0,1 b	3,8 ± 0,2 ab	8,0 ± 0,1	8,0 ± 0,1	8,3 ± 0,1	3,6 ± 0,3 a	4,7 ± 0,5 b	4,1 ± 0,3 ab	2,6 ± 0,6 a	3,7 ± 0,1 b	2,4 ± 0,4 a	6,2 ± 0,2	6,5 ± 0,2	6,1 ± 0,1	7,7 ± 0,2 a	7,9 ± 0,1 ab	8,4 ± 0,1 abc	9,0 ± 0,1	8,9 ± 0,5	9,1 ± 0,2
7	3,5 ± 0,2 a	4,1 ± 0,3 b	3,9 ± 0,3 ab	7,7 ± 0,6	8,1 ± 0,2	8,1 ± 0,1	5,3 ± 0,2	5,3 ± 0,2	5,0 ± 0,1	2,4 ± 0,6	2,8 ± 0,4	2,8 ± 0,3	6,1 ± 0,1	6,5 ± 0,5	6,5 ± 0,5	7,8 ± 0,1	8,0 ± 0,2	8,0 ± 0,2	8,6 ± 0,3	8,8 ± 0,1	8,8 ± 0,2
14	4,2 ± 0,2	4,3 ± 0,1	4,0 ± 0,2	7,7 ± 0,1 a	8,4 ± 0,2 b	8,2 ± 0,1 b	5,4 ± 0,6	5,8 ± 0,8	4,9 ± 0,2	3,2 ± 0,4	2,9 ± 0,2	3,3 ± 0,1	6,3 ± 0,5	6,3 ± 0,1	6,1 ± 0,3	8,2 ± 0,3	8,0 ± 0,2	7,9 ± 0,4	9,3 ± 0,5 a	9,0 ± 0,1 a	8,8 ± 0,0 abc
21	4,3 ± 0,1	4,2 ± 0,3	4,3 ± 0,3	8,0 ± 0,1 a	8,5 ± 0,3 b	8,2 ± 0,1 ab	5,3 ± 0,1	6,0 ± 0,3	5,9 ± 0,2	3,5 ± 0,7	3,8 ± 0,2	3,9 ± 0,3	5,3 ± 0,5	5,1 ± 0,3	5,0 ± 0,1	7,4 ± 0,1	8,0 ± 0,4	7,9 ± 0,2	9,1 ± 0,2	9,0 ± 0,5	8,7 ± 0,1
28	4,3 ± 0,1	4,6 ± 0,1	4,5 ± 0,1	8,5 ± 0,2	8,4 ± 0,1	8,4 ± 0,2	5,3 ± 0,2	6,2 ± 0,2	5,5 ± 0,3	3,1 ± 0,0	3,6 ± 0,0	3,3 ± 0,3	5,5 ± 0,3 a	4,6 ± 0,5 b	4,7 ± 0,4 b	7,7 ± 0,4	8,2 ± 0,1	7,7 ± 0,1	9,0 ± 0,1	8,8 ± 0,2	8,9 ± 0,3
35	4,1 ± 0,0	4,3 ± 0,3	4,1 ± 0,1	8,3 ± 0,5	8,1 ± 0,1	8,2 ± 0,3	5,9 ± 0,1	5,6 ± 0,3	5,8 ± 0,5	3,0 ± 0,3	2,9 ± 0,2	3,2 ± 0,6	4,2 ± 0,4	4,2 ± 0,2	4,6 ± 0,3	8,0 ± 0,8	7,8 ± 0,2	7,4 ± 0,6	9,1 ± 0,0	8,9 ± 0,3	9,0 ± 0,1
42	3,5 ± 0,5	4,0 ± 0,4	3,9 ± 0,5	8,2 ± 0,3	8,3 ± 0,1	8,3 ± 0,0	5,6 ± 0,4	4,8 ± 0,3	5,1 ± 0,5	3,0 ± 0,1	2,8 ± 0,1	3,3 ± 0,3	4,6 ± 0,7	4,2 ± 0,3	4,3 ± 0,1	7,7 ± 0,6	7,8 ± 0,0	7,8 ± 0,2	8,9 ± 0,1	8,9 ± 0,1	8,9 ± 0,1
49	3,2 ± 1,1	3,9 ± 0,1	4,0 ± 0,1	8,2 ± 0,0	8,3 ± 0,0	8,4 ± 0,1	4,9 ± 0,3	5,1 ± 0,7	5,1 ± 0,8	3,2 ± 0,3	3,7 ± 0,6	3,5 ± 0,5	4,9 ± 0,2	4,9 ± 0,4	5,1 ± 0,2	7,6 ± 0,2	7,9 ± 0,0	8,0 ± 0,1	8,8 ± 0,1	8,8 ± 0,0	8,9 ± 0,0
56	2,7 ± 0,1 a	4,0 ± 0,2 b	3,9 ± 0,1 b	7,9 ± 0,1	7,9 ± 0,0	7,7 ± 0,4	5,4 ± 0,4	5,0 ± 0,3	4,9 ± 1,3	3,2 ± 0,2	3,6 ± 0,3	3,4 ± 0,1	4,6 ± 0,2	4,6 ± 0,2	4,7 ± 0,2	7,7 ± 0,0 a	7,8 ± 0,3 a	7,0 ± 0,7 b	8,9 ± 0,1	8,7 ± 0,1	9,0 ± 0,2
63	2,6 ± 0,3 a	3,7 ± 0,3 b	3,7 ± 0,2 b	7,9 ± 0,1	7,9 ± 0,1	7,9 ± 0,2	3,7 ± 0,3	3,8 ± 1,0	4,1 ± 1,5	2,6 ± 0,4	2,4 ± 0,5	3,1 ± 0,3	4,3 ± 0,4	4,7 ± 0,1	4,1 ± 0,1	5,3 ± 0,4	5,2 ± 0,3	5,2 ± 0,3	8,6 ± 0,4	8,8 ± 0,2	8,6 ± 0,2

Average ± Standard deviation of three replicates

Values followed by different small letters in the same row are significantly different according to the Tukey's multiple comparison ($p < 0.05$)
